# Bioarchaeological investigations of the princely grave at Helmsdorf attesting to the violent death of an Early Bronze Age leader

**DOI:** 10.1038/s41598-022-20720-8

**Published:** 2022-09-27

**Authors:** Nicole Nicklisch, Frank Ramsthaler, Jan-Heinrich Bunnefeld, Georg Schulz, Ronny Friedrich, Kurt W. Alt, Harald Meller

**Affiliations:** 1 Center of Natural and Cultural Human History, Danube Private University, Förthofstraße 2, 3500 Krems-Stein, Austria; 2 Institute of Legal Medicine, Saarland University, 66421 Homburg, Saar, Germany; 3 State Office for Heritage Management and Archaeology, Saxony-Anhalt - State Museum of Prehistory, Richard-Wagner-Str. 9, 06114 Halle, Saale, Germany; 4 Department of Biomedical Engineering, University Basel, Gewerbestraße 14, 4123 Allschwil, Switzerland; 5 Curt-Engelhorn-Center Archaeometry, D6-3, 68159 Mannheim, Germany; 6 Institute of Prehistory and Archaeological Science, University of Basel, Spalenring 145, 4055 Basel, Switzerland

**Keywords:** Biological anthropology, Archaeology

## Abstract

The Helmsdorf “princely” tomb, excavated at the beginning of the twentieth century, is one of the most important archaeological discoveries dating from the Early Bronze Age in central Germany. In addition to the burial inventory, which points to an elevated social position of the deceased, a number of highly fragmented skeletal remains were preserved. Forensic anthropological investigation identified three distinctive bone defects, the surfaces of which were macromorphologically and microscopically examined in greater detail. Micro-CT analyses were also carried out. The results of all examinations suggested that the defects represented three perimortem injuries. The wound morphology was indicative of the use of a bladed weapon. The combination of injuries and their locations supported the assumption of a targeted use of force to kill. A comparison of Early Bronze Age weapons and tools with the bone lesions led to the identification of a type of weapon possibly used in the attack.

## Introduction

During the central European Early Bronze Age, metallurgy reached an unprecedented level^1^. The technical innovation of bronze production was sparked by the innovation of creating a copper and tin alloy, which would gradually replace copper and arsenic bronze as basic materials. Extensive metal production went hand in hand with mining and trading other mineral resources. The Saale-Unstrut region in southern Saxony-Anhalt was characterised by rich salt deposits and large-scale salt production^2^. Trade in salt, metal and prestige objects was facilitated by the geographical location of central Germany at the centre of European trade routes. Hoards from this period tend to contain objects such as knot-headed pins, *Ösenhalsringe*, massive arm and ankle rings, halberds and special types of flanged axes, which suggest an early form of serial production. In addition to numerous hoards, impressively constructed large burial mounds with rich grave goods also point to a highly differentiated social structure with a well-organised military might^3,4^. Prestigious finds from the late phase, such as the Nebra Sky Disc (c. 1600 BC), reflect the advanced level of knowledge of individual personalities or groups^5,6^.

The Únětice Culture was the most important Early Bronze Age cultural group in central Europe and existed in central Germany from 2200 to 1550 BC^7,8^. Its distribution extended throughout Bohemia, Moravia, Silesia, Lower Austria and central Germany. The cemetery that gave its name to the cultural group was unearthed north of Prague, in the Bohemian municipality of Únětice.

**Figure 1:**
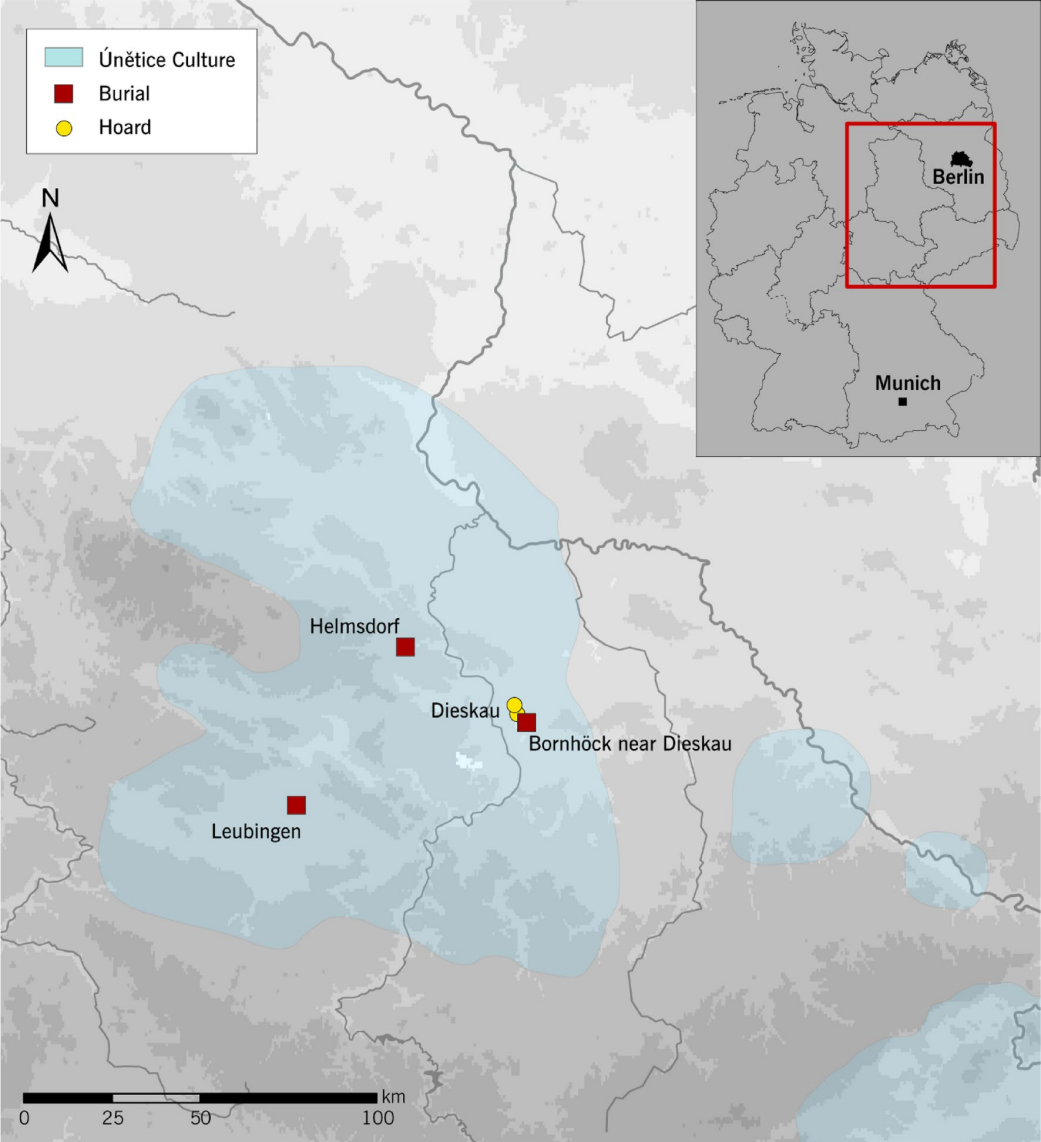
Location of the Helmsdorf burial mound, the mound at Leubingen (district of Sömmerda, Thuringia) and the so-called “Bornhöck” near Dieskau (Saalekreis district, Saxony-Anhalt) with the nearby hoards (map: Gauss–Krüger coordinate system, edited in Adobe Illustrator, State Office for Heritage Management and Archaeology Saxony-Anhalt).

Numerous rich deposits such as the hoard from Dieskau have come to light in central Germany (Fig. 1)^4,9^. Settlements were built near water courses, were unfortified and consisted of a few longhouses^10^. By far the largest known settlement was unearthed at Pömmelte^11^. Among the most important cemeteries in central Germany are Nohra and Großbrembach in Thuringia, both of which have yielded important basic data for anthropological studies^12,13^. Burials were typically flat graves (earth graves, stone cists), where the dead were uniformly interred in a crouched position on their right side, usually facing east with their head in the north. In some rare cases, stretched supine position is also found^14,15^. Double or multiple burials are not uncommon^16^.

As part of an interdisciplinary study funded by the German Research Foundation, approximately 600 skeletal finds from Saxony-Anhalt were examined bioarchaeologically, including 190 Únětice Culture burials. The osteological examinations carried out on the Únětice remains not only identified greater mean height compared to the Early Neolithic farmers, but also pointed to a more robust bone structure overall, fewer carious dental defects and a lower frequency of skeletal “stress markers”^17^. Population genetic studies show that the Únětice Culture population was primarily fed by the gene pool of local Late Neolithic groups associated with the Corded Ware and Bell Beaker cultural phenomena, which in turn took much of their genetic signature from the Eurasian steppes^18–20^. Upon arrival in central Europe in the 3^rd^ millennium BC, these groups reshaped the genetic profile of central Europeans to a significant extent. The simultaneous appearance of previously unknown pathogens was probably also connected with this wave of immigration^21^.

Of the large burial mounds from the Únětice period, usually referred to as princely graves because of their outstanding grave goods, approximately ten have been uncovered in central Germany, and only three of those have ever been scientifically studied^22^. In addition to the Helmsdorf burial under review here, these include a burial mound at Leubingen (Sömmerda district, Thuringia) and the so-called “Bornhöck” near Dieskau (Saale district, Saxony-Anhalt) (Fig. 1)^4^. At Leubingen and Helmsdorf, timber components of the burial chambers were examined dendrochronologically. The Leubingen barrow was dated to 1942 ± 10 BC^23^. Radiocarbon dating placed the Bornhöck mound in the second half of the nineteenth century BC or around 1800 BC^24^. The fact that the Helmsdorf burial mound is the only feature to have yielded skeletal remains along with the burial inventory further highlights the scientific importance of the site. For this reason, it was decided to carry out a re-assessment of the feature using modern techniques. The forensic anthropological analysis of the skeletal remains provides an insight into the possible circumstances surrounding the death of the so-called “Prince of Helmsdorf”.

## Results

### Archaeological record

**Figure 2:**
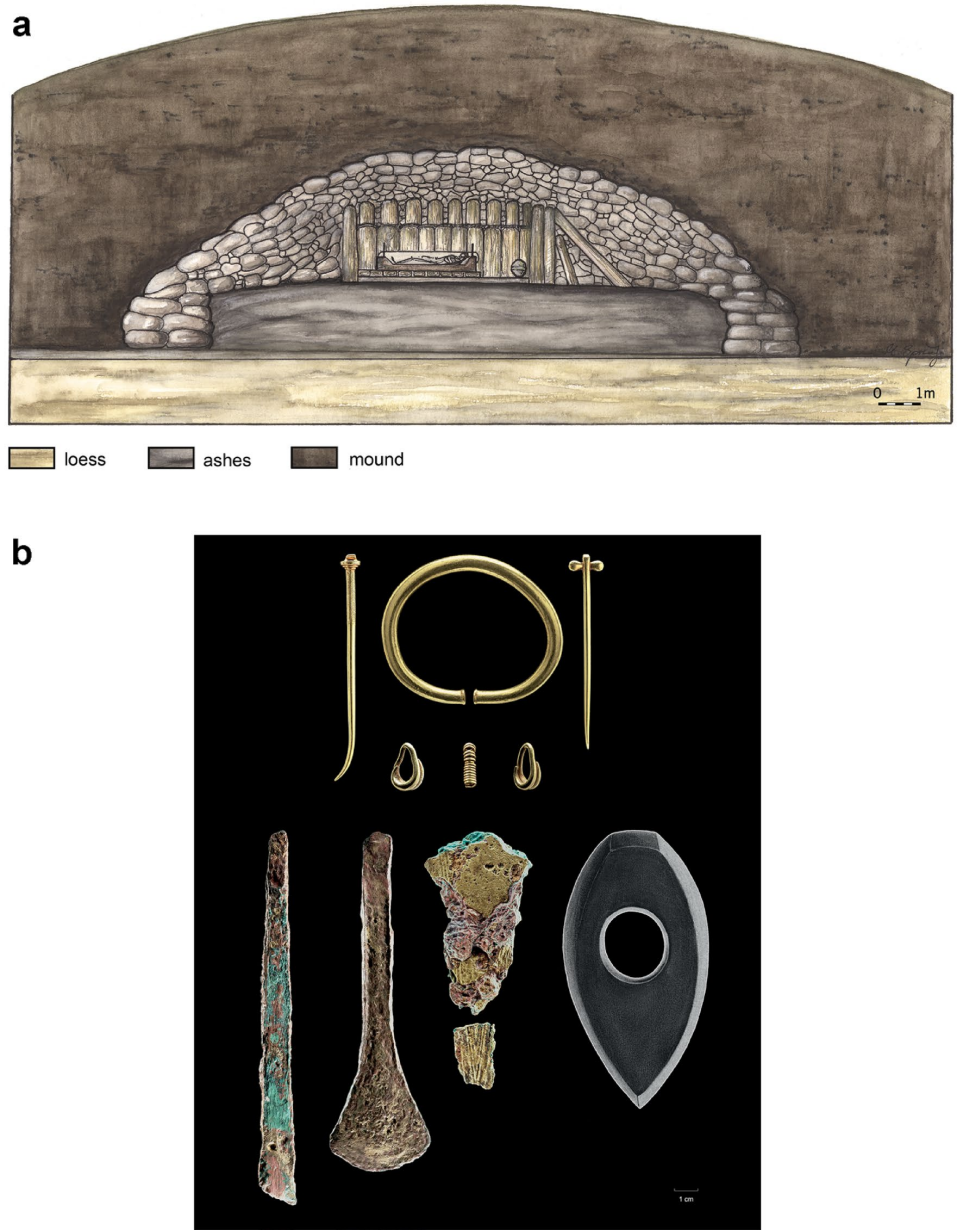
(**a**) and (**b**) Burial mound and grave goods. (**a**) Reconstruction of the Helmsdorf burial mound with the burial chamber and funerary chest (“Totenlade”) where the prince was laid out (drawing by M. Spring, State Office for Heritage Management and Archaeology Saxony-Anhalt). (**b**) The grave contained rich grave goods, including gold jewellery consisting of a massive arm ring, two pins, two lock rings and a spiral. The prince also had bronze weapons and tools, a Neolithic stone axe and a large ceramic vessel (not shown) (photo panel: J. Lipták, State Office for Heritage Management and Archaeology Saxony-Anhalt).

The skeletal remains of the Helmsdorf prince were recovered during excavations mounted in 1907. The burial mound that contained the grave was located approximately 32 km north-west of Halle (Saale) between the villages of Helmsdorf and Augsdorf (Mansfeld-Südharz district) and was also known as "Großer Galgenhügel” (great gallows hill)^25,26^. In 1906, planned construction of a railway line for the local copper slate mining industry required the removal of the hill. Since a smaller hill—the “Kleiner Galgenhügel” (small gallows hill)—had already been destroyed decades earlier, a local historian, Prof. Hermann Größler, thankfully had the foresight to initiate scientific examination of the larger hill which still existed. It was some 34.5 m wide (max. diameter west–east) and approximately 6.8 m high (average height) and contained a burial chamber built of oak wood with a central burial (Fig. 2A).

The few preserved skeletal remains were dark brown to greyish-black in colour and showed clear traces of weathering, which can be attributed, among other things, to the fact that the body had been laid out on a funerary chest made of oak, which measured approximately 2 m in length and 98 cm in width. It was also reported that not only the bones, but the entire contents of the funerary chest, were covered by “light, loose ash or ashy earth”^25^ (p. 22). The excavator suspected that the ash had originated from a sacrificial fire lit above the gable of the burial chamber, which, after the collapse of the roof construction, had been mixed with the earth inside the grave^25^. The position of the skeleton on the funerary chest, which was found south-north-aligned, is not entirely certain; it may have lain in a stretched supine position or in a crouching position; there is certainty, however, regarding the position of the head, which pointed north and faced east^25,26^. Numerous gold, bronze and diorite grave goods were found in the chest area of the deceased (Fig. 2B). Some sherds of a ceramic vessel were also recovered from the burial chamber. In addition to the central burial, the barrow also contained the skeletal remains of four other individuals, which were interpreted by the excavator as human sacrifices; some of them were buried in stone cists either before or after the sacrificial fire^25^. More recent results have shown that the prince's grave had overlain two burials of the Corded Ware Culture, which refutes the human sacrifice theory. Other burials from the period found in the vicinity of the mound pointed to a continuous use of the site as a burial ground from the Late Neolithic to the Early Bronze Age^27^.

Initial radiocarbon dating carried out on the bones in 2014 resulted in a calibrated date of between 2106 and 1894 cal BC (2σ, MAMS 19418, ^14^C-age = 3616 ± 27 yr BP). Further analysis undertaken in 2017 yielded a date of between 2287 and 2066 cal BC (2 σ, MAMS 30954, ^14^C-age = 3773 ± 24 yr BP). The relatively large difference between the two radiocarbon dates remains unexplained. Bone preservation was excellent in both cases with 5% and 8% collagen content and CN-ratios of 3.4 and 3.2, respectively, indicating good quality collagen. Radiocarbon dating on human bone does not necessarily reflect the date of death. Carbon is incorporated into the bone structure during the period of major bone growth in the first two decades of life. After that point, carbon is constantly replaced at a rate of a few percent per year. Thus, bone contains a mixture of carbon originating from the entire lifespan—known as the “human bone collagen offset” (HBCO)^28^. Using the anthropologically determined age at death of 30–50 years (see the next chapter) an HBCO correction of around 20 ± 10 years was applied, resulting in calibrated ages of 2119-1785 cal BC and 2286-2038 cal BC, respectively. Much more precise dating can be gleaned from the dendrochronological data derived from the oak wood of the funerary chest in 2017, which dates the find to 1829/1828 BC (CEZ Mannheim, MAD 1479 and MAD 1480). This result is in good agreement with the first radiocarbon date.

In addition to the monumental burial and the high-quality grave goods, the results of the nutritional reconstruction using the stable isotopes carbon (δ^13^C) and nitrogen (δ^15^N) also proved helpful in reconstructing the social status of the deceased^29^. The nitrogen isotope ratios showed that the elevated social status of the Helmsdorf prince seen in the burial context was further supported by the dietary habits of a privileged lifestyle, characterised by preferential access to animal protein (δ^15^N = 12.5 ‰ vs. 10.4 ± 0.5 ‰ for non-elite burials, and 7.3 ± 0.8 ‰ for domestic herbivores)^29^.

### Anthropological record

**Figure 3:**
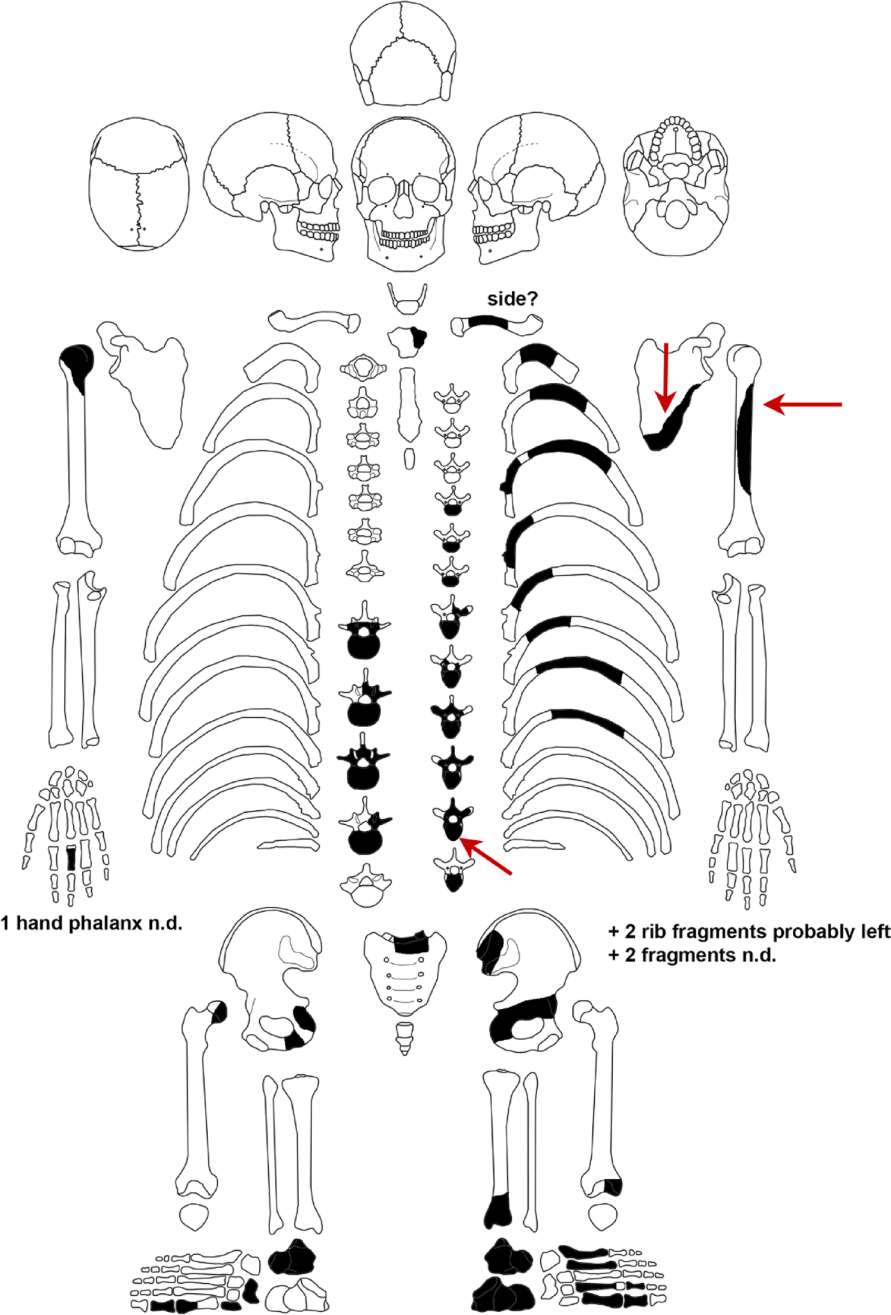
The preserved skeletal remains from the Helmsdorf burial. The bone injuries are marked by arrows. In some of the bone fragments, the side of the body from which they came from could not be determined (n.d.) (image by N. Nicklisch).

The skeletal finds mainly represent the area of the trunk and include thoracic and lumbar vertebrae, rib fragments, the pelvis (including the right acetabulum) and the left scapula as well as the adjacent extremities (the right humeral and femoral heads) (Fig. 3). Diaphyseal remains from the left humerus, part of the left distal tibial epiphysis as well as a hand phalange (side unclear) and some foot bones are also present. The excavation report by Größler^25^ shows that essential skeletal elements were already missing when the feature was uncovered. However, smaller fragments of the skull and a jawbone fragment with teeth are also mentioned, which have since been lost.

The poor state of preservation of the Helmsdorf skeleton compared to other, very detailed osteobiographies of Early Bronze Age burials^30,31^ clearly limits the data collection. The size and structure of the bones point to an adult individual. As the epiphyses of the humerus and femur are already completely fused and no fusion lines are visible, we can assume that the individual was over 25 years old. Slight degenerative changes in the vertebrae and joints indicate regular physical strain, although the individual’s personal predisposition should not be underestimated. In contrast to the vertebrae, the signs of wear are minimal on the humeral head and moderate on the glenoid cavity, which in itself is not a very reliable age marker. Given the limited findings, the individual’s age at death can only be narrowed down very roughly to between 30 and 50 years.

The metric examinations of the humeral head (vertical diameter: 43.6 mm) and calcaneus (greatest length: approx. 80 mm) are indicative of a male individual. The pronounced muscle attachment on the humerus (Tub. deltoidea, left), the thickness of the Tuber ischiadicum (left) and the size of the acetabulum (left) also support the diagnosis "male". Unfortunately, several attempts to determine the biological sex by genetic means proved unsuccessful. In summary, the results of the anthropological analysis suggest that these are the skeletal remains of a man about 30–50 years old.

### Palaeopathological findings and anatomical features

The thoracic and lumbar vertebrae show arthritic changes in the form of spondylosis deformans and spondylarthrosis deformans. In addition, local endplate herniations can be seen on two lumbar vertebrae, which have led to a depression of the nucleus pulposus and are known as Schmorl nodes^32^. Such vertical herniations can result from excessive biomechanical stress such as physical activity, from obesity or from metabolic disease or Scheuermann's disease^33–35^. The size and shape of the vertebral compartments apparently play a role in the development of Schmorl nodes^36^. The 10th thoracic vertebra exhibits another distinctive feature: here, a "deformation" of the vertebral body with a lateral inclination of the processus spinosus indicates slight scoliosis, which is also evident in the adjacent vertebrae.

A squatting facet is visible at the left distal end of the tibia. This activity marker results from regular sustained pressure on the upper ankle joint due to, for example, frequent squatting or kneeling^37,38^, and is very common in prehistoric populations.

### Evidence of perimortal violence

**Figure 4:**
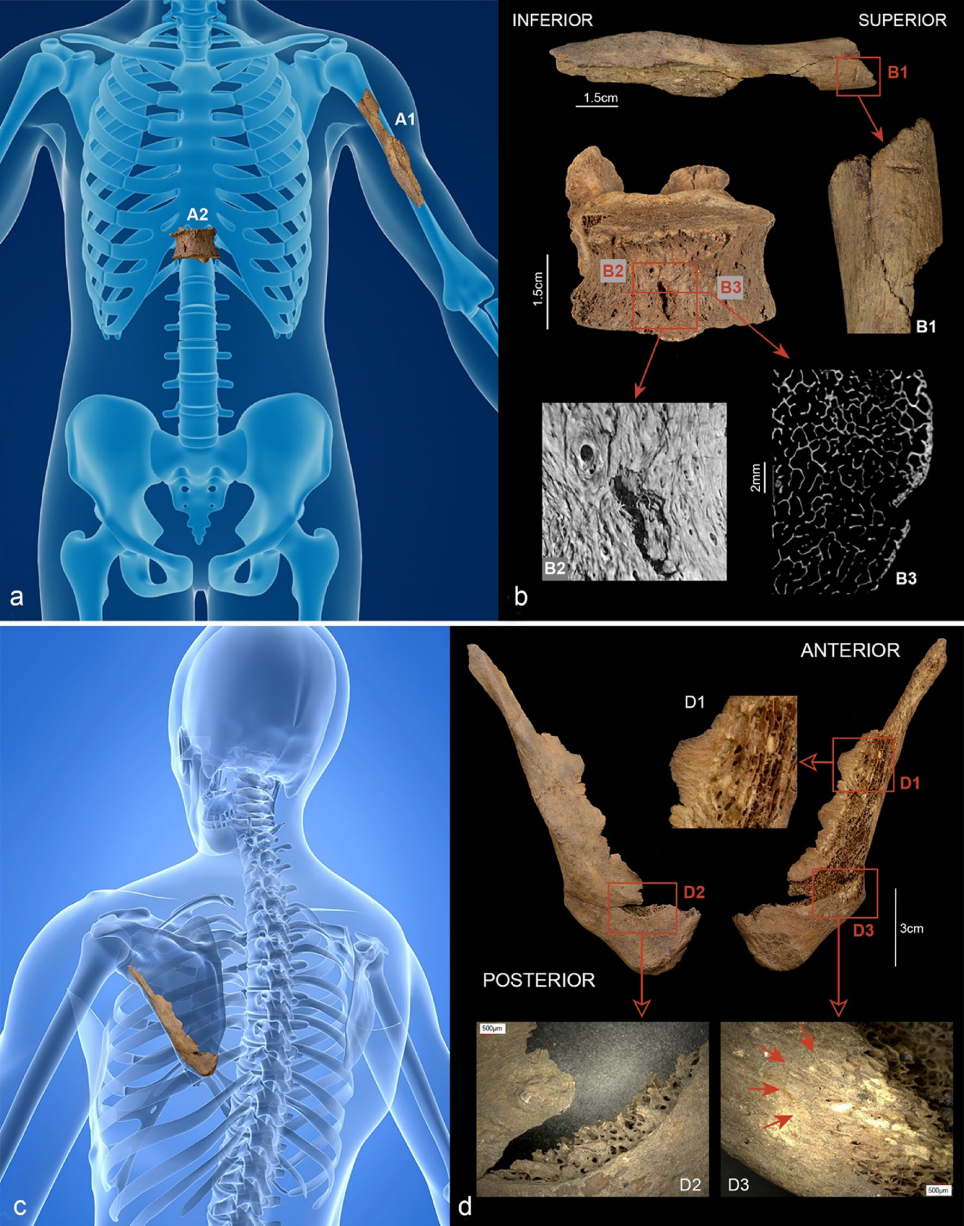
(**a**–**d**) Reconstructed bone injuries. (**a** and **b**) The injuries to the left humerus (**A1**) and the 11th vertebral body (**A2**). (**A1**) and (**B1**) show a kerf in the upper third of the left ventro-lateral humeral diaphysis. The defect, which runs transversely to the longitudinal axis, is 4.9 mm long and shows a V-shaped profile. (**A2**) and (**B2**) show the 11th thoracic vertebra which bears a slit-like ventral defect measuring approx. 6 mm in length. The micro-CT images show a terraced depression with small hairline cracks on the left side of the defect (**B2**); in the transverse section (**B3**) the lesion in the vertebral body reaches a depth of approx. 3 mm. (**c** and **d**) Defect on the left scapula. The bone is dissected by a hard tissue defect approx. 120 mm long with bevelled edges (**D1**). A crescent-shaped defect is visible above the inferior angle of the scapula (**D2**). The bone surface appears peeled in some areas (**D3**) (photos: N. Nicklisch, G. Schulz, (**a**) Background: A. Ciprian/Shutterstock.com modified by N. Nicklisch, (**c**) Background: SciePro/Shutterstock.com modified by N. Nicklisch).

Three lesions on the skeleton raise the possibility of a violent origin. One is a bone defect in the shape of a notch in the upper third of the left ventro-lateral humeral diaphysis (Fig. 4A1,B1). The defect, which runs at right angles to the bone axis, is 4.9 mm long and has a symmetrical V-shaped section with discrete marginal lesions.

A slit-like defect measuring approximately 6 mm in length and oriented in an 11 o’clock position can be seen on the anterior/ventral side of the 11th thoracic vertebra, slightly to the right of the midline (Fig. 4A2,B2). According to micro-CT images, it penetrates about 3 mm into the vertebral body (Fig. 4B3, bottom right). The lesion shows a terraced indentation on the right side with two small hairline fissures branching off at the upper edge (Fig. 4B2 bottom left).

The third defect is located on the left upper shoulder, is approx.120 mm long and penetrates the scapula at an angle. The edges of this linear lesion are characterised by diffuse bevelling (Fig. 4C,D1). A crescent-shaped defect is visible just above the inferior angle of the scapula; it was probably caused by the point of the blade breaking through the bone surface (Fig. 4D2). In some marginal areas, the bone surface appears peeled (Fig. 4D3).

No new bone formation can be identified in any of the three defects.

## Discussion

The question of whether these defects are signs of violence or whether they represent taphonomic processes or damage from the recovery of the bones during the excavation is primarily determined by the fact that they are linear and short, as is characteristic of a sharp impact with a pointed object^39,40^. In view of the isolated nature of the injuries, a postmortem manipulation of the bones while they were still covered in soft tissue seems unlikely. Relevant features in this respect are the bevelling and peeling of the bone surface of the scapula, the terraced depressions and hairline cracks on the vertebral body and the V-shaped section of the defect on the humerus^41,42^. Such changes are uncharacteristic of postmortem taphonomic processes on dry bone^43,44^. In addition, there is no evidence of healing in any of the three injuries. All defect patterns thus point to a perimortal origin.

To answer the question of what type of weapon was most likely to have caused the injuries, we must take into account not only the biomechanical texture of the bone areas in question, but also the physical properties of the weapons under consideration. The weapons that may have been used in nineteenth century BC central Germany are axes, halberds and daggers. Spearheads and swords were not common in the region at the time, and did not become widespread in central Europe until later: spearheads from the eighteenth century BC, and swords from the seventeenth century BC^45,46^.

The injury to the 11th thoracic vertebra shows the acute angles typical of dagger stab wounds (cf. Fig. 4B2). Contrary to knives, which have a blunt edge or back, daggers have double-edged blades^39^. In this case, subtle differences can be seen between the two short, slit-like lesions on the vertebra and left humerus (cf. Fig. 4B1,B2). This discrepancy can be explained by the differences in bone structure (compact bone/cancellous bone) combined with the angle at which the weapon entered the bone, and should most certainly not be interpreted as evidence of two different weapons. If a sharp, pointed blade gets stuck in cancellous bone and can only be removed with considerable force, e.g. by shaking or pulling it out, so-called exit injuries accompanied by secondary cracks can occur^40,44^. This is also consistent with the wound morphology of the scapula. Thus, all three injuries can be explained by the use of a dagger as a weapon.

In this context, experimental studies by Downing and Fibiger^47^ might be of interest, where replicas of Bronze Age weapons were tested on bone-like polyurethane models (Synbone). As well as an axe and a sword, a dagger was also tested. The blade of the dagger was damaged during the first contact with the artificial bone model. Although Downing and Fibiger^47^ expressed doubt concerning the suitability of artificial bone for reconstructing sharp force trauma, the experiments undoubtedly raised questions regarding the usability and longevity of bronze weapons in combat, particularly in terms of the hardness and durability of the blades. Of the three injuries presented here, this appears to apply most to the injury to the scapula, because this is where any damage to the blade is most likely to have occurred, either during the impact or during the extraction of the weapon.

**Figure 5:**
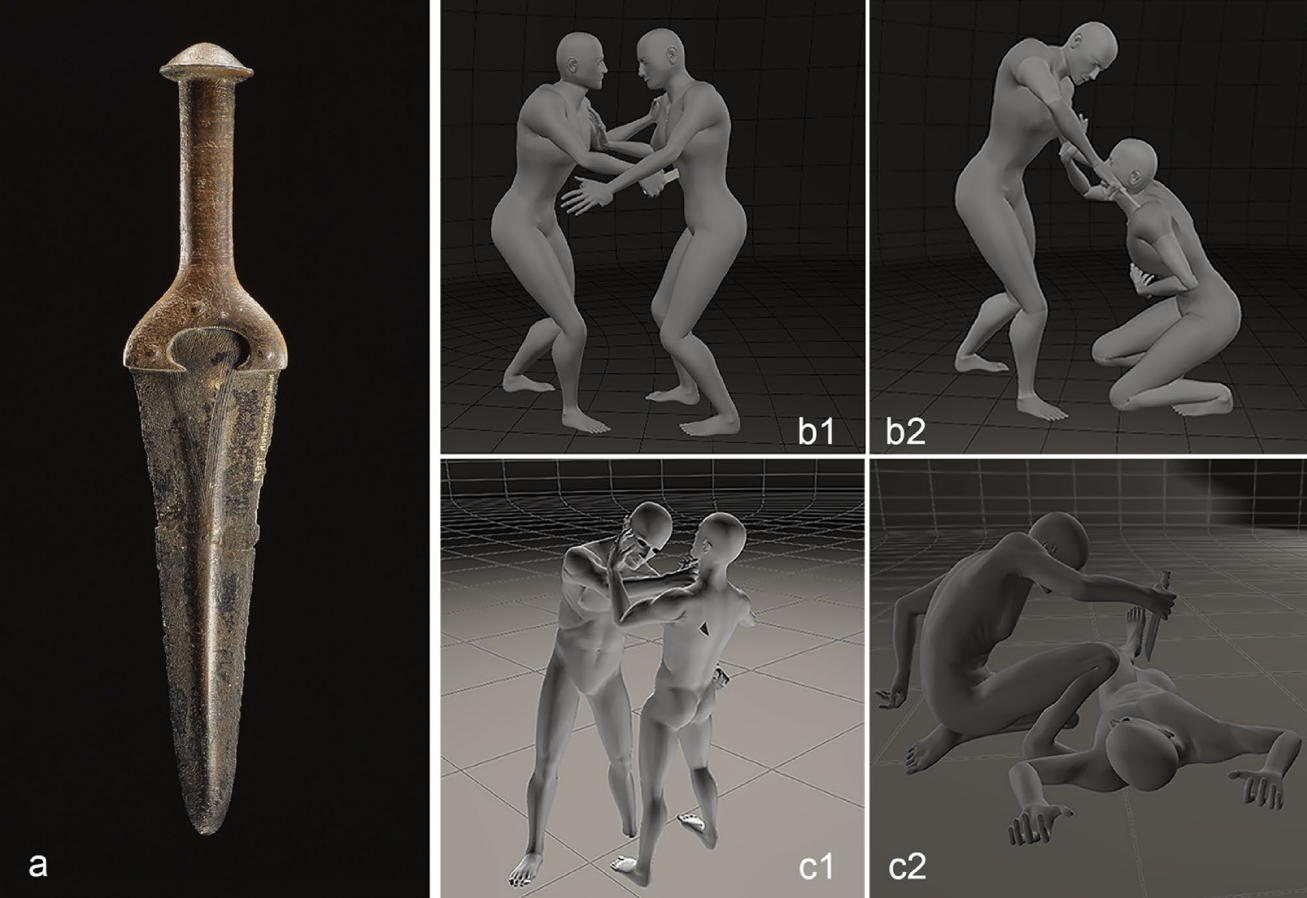
(**a**–**c**) Type of weapon potentially used and possible sequence of the attack. (**a**) The blade of the Giebichenstein dagger measures 17.4 cm in length and is a typical example of a Únětice Culture metal-hilted dagger. The blades of these daggers are generally between 17 and 22 cm long (photo: J. Lipták, State Office for Heritage Management and Archaeology Saxony-Anhalt). (**b** and **c**) Different sequences of events are possible. Two scenarios are described as follows: Scenario b: the stabbing in the abdomen occurred first (**b1**); the stab wound to the shoulder was inflicted when the victim was on his knees or bent over (**b2**). Scenario c: the victim was stabbed in the shoulder first (**c1**) and then in the abdomen when he was already lying on the ground (**c2**). The injury to the humerus may have occurred early-on in the attack, when the victim was still able to defend himself (images prepared using Poser 3D graphics by N. Nicklisch and F. Ramsthaler).

Based on comparisons with other artefacts and the current state of research, a metal-hilted dagger as shown in Fig. 5 is the type of weapon most likely to have been used. The metal-hilted dagger from Halle-Giebichenstein (Fig. 5A) has a metal hilt and a blade measuring 17.4 cm in length^48^ (p. 46 No. 4). The blades of this "Únětice type" dagger are usually between 17 and 22 cm long, but some metal-hilted dagger types have longer and wider blades; the latter have a different focus of distribution^48^. Dagger blades with organic handles (so-called hilt-plated daggers) are also known from eastern Germany, although they tend to be somewhat shorter there. The halberd, which is characteristic of the Early Bronze Age, and which, apart from its status as a ritual object or status symbol^49^, was most likely used as a cutting or slashing weapon can be ruled out in this case. Although double-bladed, the slashing motion would result in a striking angle that is not consistent with the stab wound morphology observed. This has also been shown in experimental studies^50^. The same applies to the axe (wedge-shaped blade), which is classified as a chopping weapon and would cause significant fracturing and wastage of bone^47^.

Pulling out a blade at a different angle or the victim moving when being stabbed can leave a swallow-tail wound pattern in soft tissue. In abdominal injuries, the blade must cover a distance of about 25–30 cm from the surface of the abdomen to the anterior edge of the vertebra (in a medium-sized body), though it is not always possible to determine the length of the blade from this. Since the abdominal wall is very often pushed inwards during the stabbing, the stab wound can be significantly longer than the blade itself^51^. With this in mind, we can say that the blade used here could not have been any shorter than approx. 17–18 cm. The metal-hilted dagger from Halle Giebichenstein, whose blade measured 17.4 cm, and most other daggers of the "Únětice type" would certainly meet these criteria.

In the case of abdominal stabbing from the front, a blade penetrates several internal organs, and the severity of the injuries can be deduced from the nature of the blade. In this case, the stab wound would have been life-threatening due to the penetration of the liver, the abdominal aorta (approx. 3 cm in diameter) and the great vena cava (approx. 2.5 cm in diameter) running along the front of the spinal column. Based on the direction of the defect on the vertebral body, we can assume that the blade penetrated the body in the area of the aorta, inflicting a puncture injury in the hollow organ, which would have resulted in rapid bleeding into the posterior abdominal cavity (retroperitoneal space). Though we cannot necessarily assume that the victim was immediately incapacitated, the injury would ultimately have been fatal, particularly because of inadequate medical care in a prehistoric context.

Sharp force fatalities are usually associated with multiple injuries^52^. The stabbing sequence can rarely be determined in retrospect. In case of the Helmsdorf prince, the traces of various sharp-force impacts suggest a sequence of events; however, it must be pointed out that the actual number of injuries and potential injury clusters remain unclear due to the incomplete preservation of the skeletal remains and the absence of soft tissue.

From the point of view of reconstructing the events, the injuries to the left scapula and the 11^th^ vertebra allow for more than one interpretation (Fig. 5, scene B and C). The damage to the scapula with its strongly bevelled, almost tangential damage pattern is consistent with a stabbing from above with the opponent standing upright and wielding the blade with their arm outstretched (Fig. 5C1). However, it is just as plausible that the victim was in a kneeling position with the attacker standing upright (Fig. 5B2). Regardless of which scenario actually occurred, we can assume that there was an injury to the jugular vein, which, in addition to the blood loss, may have led to a so-called air embolism and this would have quickly initiated right heart failure^39^. Whether the stab wound to the abdomen represents the final event (cf. Fig. 5C2) or whether it was delivered before the other injuries, cannot be ascertained based on the injury morphology. In the case of the shoulder injury, we can expect the blade to have been damaged. Therefore, it seems more plausible for the stab wound to the abdomen to have been inflicted first and the injury to the shoulder last (cf. Fig. 5 scene B1–B2). Nevertheless, any attempt at reconstructing the sequence of events must remain hypothetical.

In injuries from knives or similar objects, active stabbing is usually necessary to cause deep injury, as clothing and the outer layer of skin offer considerable resistance. The fact that bony lesions were present also proves that the attack was rather violent.

In contrast to swords, knife-like weapons such as the dagger in question are generally light-weight and short; in view of the depth of injury identified here, this means that the attack was violent and occurred at close quarters. It was the transfer of the attacker's weight onto the weapon that caused the severity of the injury in the first place. Studies have shown that less than 10% of all fatal injuries are caused by sharp force to the abdominal area^53^. Survivors of such attacks report initially perceiving the stabbing as a blow and only later recognise it as a stab wound because of the blood.

Armour, mainly to protect the chest, is known to have been used in the Late Bronze Age (from c. 1300 BC), and helmets and greaves were also worn^54^. It is, of course, possible that armour made of an organic material such as leather already existed in the Early Bronze Age or even earlier. The design found on a stele at Petit-Chasseur, Switzerland, may represent such armour^55^ (p. 83). Finds of damaged and repaired cuirasses show that even metal armour does not offer one hundred percent protection^54^ (pp. 207–209). We can assume, however, that experienced fighters mainly targeted body parts that—despite the use of armour—offered access to important blood vessels or life-sustaining organs in a bid to induce maximum blood loss and rapid incapacitation^56^. The neck, shoulder and abdomen are such areas. Injuries to these are well documented in archaeological bone finds, with some even including fragments or points of weapons^41,42,57–59^. Recent studies of sharp force injuries have revealed defects to bone in 66% of the cases, most of which (57%) were found on the torso^52^.

The morphology of the abdominal wound might suggest that the Helmsdorf prince was not wearing any protective equipment when he was killed and, in consequence, we could speculate that the attack probably did not occur in the context of a military conflict^60^. This would mean that the attack took place ‘in private’ and thus potentially by a person in the prince's own environment. However, due to his assumed supreme social status and leading position, a violent conflict in the context of warfare is also conceivable. So far, no large-scale battles are known to have taken place in the central European Early Bronze Age, but smaller conflicts cannot be ruled out, especially those necessary to maintain leadership^3,61^. It is possible, therefore, that the prince was fatally injured in combat. Needham et al.^30^ (p. 48–49) discuss the use of daggers as weapons for a particular type of combat in the context of a codified confrontation among members of the earliest Bronze Age elite in Britain. According to the authors, leadership could have been fought out through interpersonal combat. Both the "murder theory" and the idea of a "codified combat-contested leadership"^30^ (p. 49) remain speculative but raise interesting questions with regard to the establishment and maintenance of leadership in the Early Bronze Age. In the case of the Prince of Helmsdorf we can conclude that the combined injuries attest to a targeted use of massive force, which speaks to the perpetrator's intention to kill and to their familiarity with the combat techniques required.

## Methods

Age and sex determination is usually guided by standardised sets of methods found in the established literature^62–64^. However, due to the incomplete preservation of the skeleton, the methodological applications and statements were rather limited. In the absence of certain characteristics, age assessment was therefore primarily based on skeletal maturity and the degenerative changes observed in the vertebral bodies and joint ends. Inferences about sex were drawn from a number of metric determinations that could be collected from the humeral head and calcaneus^65–67^. For comparison of the vertical diameter of the caput humeri, regional data from adult skeletal Únětice Culture burials can be used (mean value for male individuals (n = 21): 44.4 mm; mean value for female individuals (n = 23): 40.95 mm)^17^.

From a forensic point of view, blunt and sharp force trauma or gunshot wounds can be differentiated on the basis of the type of violence involved, both in recent victims and in archaeological skeletal finds^39,68–72^. Sharp force trauma as caused by sharp knife blades, swords or axes is attested to by characteristic puncture wounds or cut and chop marks^39,71,73^. Important features are the section, width, depth and length of the injury as well as possible stripes or grooves on the wound edges, which can help to identify the weapon. The wound spectrum varies from superficial hard tissue defects to completely severed bone parts, depending on the type of weapon used, and how it was deployed or the force applied. Fracture lines may extend from the primary cut or puncture site. These characteristic features may be superimposed by taphonomic/postmortem processes^43,74^.

The assessment of the palaeopathological changes to the bones was carried out macroscopically, using a magnifying glass (magnification factor 3) and by scattered light and transmitted light microscopy (Keyence, VHX 700F). The images for the reconstruction of the possible course of events (Fig. 5 scenes B and C) were produced using Poser 3D graphics software (Bondware Inc.). To better represent the localisation of the bone defect, two Shutterstock images were acquired (Fig. 4A,C).

### Micro-CT imaging

For differential diagnosis the 11^th^ thoracic vertebra was analysed by X-ray microtomography at the Core Facility Micro- and Nanotomography (MiNa) at the University of Basel. The data were acquired by means of a nanotom m (phoenix|x-ray, Waygate Technologies, Wunstorf, Germany) which is equipped with a 180 kV/15 W nanofocus X-ray source. For the present study, an acceleration voltage of 90 kV, and a beam current of 200 µA was used. The mean photon energy was increased by adding a 0.25 mm Cu filter. The effective pixel size was set to 30 μm and the exposure time per radiograph to 3.75 s. A total of 1700 equiangular radiographs were recorded over the angular range of 360°. The projections were reconstructed using a cone beam filtered back-projection algorithm using phoenix datos|× 2.0.1—RTM (Waygate Technologies,Wunstorf, Germany). VGStudio MAX 2.1 (Volume Graphics GmbH, Heidelberg, Germany) was used for the visualisation.

### Radiocarbon dating

Both radiocarbon-dated samples were analysed at the Curt-Engelhorn-Center Archaeometry in Mannheim^75^. Collagen was extracted from the samples using a modified version of the Longin extraction protocol^76^ which includes ultrafiltration in order to remove short-chained contaminations. The collagen was then combusted in an elemental analyser (Elementar Analysesysteme GmbH) and the resulting CO_2_ reduced to graphite using a commercially available graphitization unit (AGE3, IonPlus AG). A MICADAS-type Accelerator-Mass-Spectrometer (AMS) was used for radiocarbon determination. Calendar-age calibration was performed using the software OxCal 4.2^77^ and the calibration dataset IntCal13^78^.

### Dendrochronology

Four samples of oak wood from the funerary chest were examined in 2017 using CT images at the Klaus Tschira Archaeometry Center at the University of Heidelberg and the Curt-Engelhorn-Center Archaeometry in Mannheim and then compared with the regional oak chronology (MAD 1479, MAD 1480, MAD 1481, MAD 1482). The samples comprised 58–187 annual rings, with two samples including wane and thus providing dates to the year (MAD 1479, MAD 1480).

## Data Availability

All data are included in the manuscript. The skeletal remains are currently housed at the State Office for Heritage Management and Archaeology Saxony-Anhalt in Halle (Saale), and are the property of Lutherstadt Eisleben, Regionalgeschichtliche Sammlungen.
